# Preoperative factors associated with quadriceps strength 1 year after total knee arthroplasty: a prospective longitudinal study

**DOI:** 10.1186/s12891-026-10657-8

**Published:** 2026-10-21

**Authors:** Per Sjöström, Lena Nordeman, Ola Rolfson, Anette Larsson

**Affiliations:** 1 Department of Health and Rehabilitation, Institute of Neuroscience and Physiology, Sahlgrenska Academy, University of Gothenburg, Gothenburg, Sweden; 2 Region Västra Götaland, Närhälsan Rehabilitation Centres, Lerum, Sweden; 3 Education, Development and Innovation, Region Västra Götaland, Primary Health Care, Södra Älvsborg, Research, Borås, Sweden; 4 Department of Orthopaedics, Institute of Clinical Sciences, Sahlgrenska Academy, University of Gothenburg, Gothenburg, Sweden; 5 Orthopaedics, Sahlgrenska University Hospital, Gothenburg, Sweden; 6 General Practice/Family Medicine, School of Public Health and Community Medicine, Institute of Medicine, Sahlgrenska Academy, University of Gothenburg, Gothenburg, Sweden

**Keywords:** Total knee arthroplasty, Quadriceps muscle, Muscle strength, Physical therapy specialty, Muscle strength dynamometer, Longitudinal studies.

## Abstract

**Background:**

Quadriceps strength is highly related to physical function following total knee arthroplasty (TKA). Preoperative factors associated with long-term postoperative strength remain insufficiently understood. The aim of this exploratory study was to investigate preoperative factors associated with normalized quadriceps strength (NQS), changes in NQS and limb symmetry index (LSI) 1 year after TKA in patients with uncomplicated recovery.

**Methods:**

A prospective longitudinal study was conducted. Measurements took place at three primary care rehabilitation facilities in Region Västra Götaland, Sweden. The final analysis included 39 participants aged 65 years or older who underwent elective fast-track primary TKA due to knee osteoarthritis and had an uncomplicated recovery. Preoperative factors associated with NQS in the operated limb after 1 year (*n* = 39), changes in NQS from preoperative testing to 1 year after TKA (*n* = 39), and LSI (*n* = 35) were analyzed in three separate exploratory multiple linear regression models. Preoperative factors included age, sex, NQS, the 6-minute walk test (6MWT), and the LSI.

**Results:**

Preoperative NQS (*B* = 0.72, 95% CI: 0.40 to 1.0, *p* < 0.001) and LSI (*B* = − 0.17, 95% CI: − 0.29 to − 0.037, *p* = 0.013) were significantly associated with NQS 1 year after TKA. LSI was the only preoperative factor significantly associated with changes in NQS (*B* = − 0.19, 95% CI: − 0.32 to − 0.059, *p* = 0.006) and LSI 1 year after TKA (*B* = 0.41, 95% CI: 0.15 to 0.68, *p* = 0.004).

**Conclusions:**

In this selected and relatively small sample, greater normalized quadriceps strength in the operated limb and higher LSI 1 year after TKA appeared to be primarily associated with higher preoperative normalized quadriceps strength in the operated limb and higher preoperative LSI, respectively, whereas strength gains appeared to be associated with lower preoperative LSI. The findings should be considered exploratory and primarily applicable to older adults with uncomplicated recovery after TKA. Confirmation in larger studies is warranted.

**Trial registration:**

Retrospectively registered February 9, 2022 (http://clinicaltrials.gov, ID: NCT05248815).

**Supplementary Information:**

The online version contains supplementary material available at https://doi.org/10.1186/s12891-026-10657-8.

## Background

Quadriceps strength is fundamental for optimal knee function and is strongly related to physical function after total knee arthroplasty (TKA) [1–3]. Patients undergoing TKA due to knee osteoarthritis (KOA) generally have weaker quadriceps strength compared with age-matched controls [4, 5] and are weaker in the operated limb than the contralateral limb before and after surgery [6]. These findings highlight the importance of understanding the preoperative factors associated with quadriceps strength after TKA. To date, only a limited number of studies have addressed this issue, with inconclusive results [7–9].

Proposed mechanisms of quadriceps weakness in patients with KOA are disuse due to pain [10] and arthrogenic joint inhibition [11]. To counteract these mechanisms, strengthening exercises are a core component of non-pharmacological and non-surgical treatment [12, 13]; together with maintaining physical activity it may help preserve physical function [14]. Preoperative quadriceps strength predicts physical function after TKA [15, 16]. After TKA, quadriceps strength initially decreases and then gradually increases from 3 to 6 months up to 2 years after surgery [8]. Improving quadriceps strength is an important aim of rehabilitation protocols after TKA, although the optimal exercise strategy remains unclear [17]. Normalized quadriceps strength (NQS), defined as quadriceps strength relative to body weight, is strongly associated with physical function after TKA [1].

The influences of sex, body mass index (BMI), and age on postoperative quadriceps strength have been studied, with inconsistent results [7–9]. Women had less strength loss in one study [7], whereas women and individuals with higher BMI had lower strength gains in another [9]. In a meta-regression analysis on effect modifiers across studies, the association between postoperative quadriceps strength and sex, BMI, and age varied in terms of significance and direction across follow-up periods [8].

Based on trajectories for NQS in the operated limb, preoperative values seem to be associated with postoperative values, as better preoperative values may provide a better starting point [2, 18]. Walking capacity, as measured with the 6-minute walk test (6MWT) [19, 20], may provide complementary information to strength testing, as it reflects the combined effects of lower-limb muscle strength, pain, neuromuscular control, and cardiopulmonary fitness. Additionally, the preoperative interlimb quadriceps strength ratio, as measured with the limb symmetry index (LSI) [21], could indicate a potential gap for postoperative strength gains. It is possible that greater strength asymmetry reflects altered limb-loading and pain-related or arthrogenic muscle inhibition in the operated limb.

Previous studies have been limited by the absence of objective measures of physical function or limited follow-up time [7–9]. The aim of this exploratory study was to investigate preoperative factors associated with NQS, changes in NQS and LSI 1 year after TKA in patients with uncomplicated recovery. This study extends previous work by simultaneously including demographic factors and objective measures of physical function (i.e., preoperative NQS, LSI, and 6MWT) and by investigating their associations with NQS outcomes at 1 year after TKA.

## Methods

### Study design and setting

This prospective longitudinal study was approved by the Swedish Ethical Review Authority (Dnr 2021–03590 and 2024-04643-02) and was retrospectively registered (February 9, 2022) in the Clinical Trials Registry (ID NCT05248815) as prospective registration was unintentionally not completed before enrollment of participants commenced. The reporting of this study was guided by the Strengthening the Reporting of Observational Studies in Epidemiology statement [22]. The regression analyses were developed after data collection had been completed. All participants provided written informed consent before testing. Participants were recruited by the registering nurse while being given preoperative information at Alingsås Hospital (Alingsås, Sweden)—a facility that uses fast-track care, as defined in a previous study [23]. Participant recruitment and screening were conducted between November 1, 2021, and November 7, 2022. Data collection continued until November 2023 and was conducted at three primary care rehabilitation facilities in Region Västra Götaland, Sweden. Data collection and baseline testing were performed 1–14 days before surgery, and postoperative testing was performed 1 year after surgery. All data were collected by the project leader (PS). Consequently, the assessor performing the follow-up measurements was not blinded to participants’ baseline results.

### Participants

Participants scheduled to undergo elective primary TKA due to KOA were asked to participate after receiving oral and written information about the study. The inclusion criteria were as follows: 65 years of age or older, elective primary TKA due to KOA, and expected to participate in primary care rehabilitation. The exclusion criteria were inability to read or speak Swedish and inability to perform strength testing. At postoperative testing, participants were excluded from the final analysis if they were unable to complete strength testing or if events occurred that were considered likely to impact their recovery of quadriceps strength (i.e., complications after TKA or other medical conditions, treatments, or surgeries). All participants received the same guideline-based standard care rehabilitation, as previously described [18]. In short, follow-up appointments were scheduled at 1, 3, and 6 weeks postoperatively; from 6 weeks onward, treatment was individually tailored. The authors were not involved in the rehabilitation or surgical procedure of any of the participants.

### Variables

#### Outcomes

The outcome (dependent) variables were (model 1) NQS in the operated limb 1 year after TKA, (model 2) change in NQS in the operated limb from preoperative assessment to 1 year after TKA, and (model 3) LSI of NQS 1 year after TKA. Change in NQS was included as an outcome because the magnitude of improvement in NQS from baseline to 1 year after TKA was considered clinically relevant and conceptually distinct from absolute postoperative NQS at 1 year. NQS was calculated as a percentage of body weight, given by the following formula: maximum voluntary isometric contraction (newton [N]) / body weight (N) * 100. Changes in NQS were calculated as NQS at 1 year minus preoperative NQS, with positive values indicating improvement. The LSI as a percentage was calculated using the following formula: NQS in the operated limb/NQS in the contralateral limb * 100. Due to their shared mathematical components, associations involving both NQS and LSI may partly reflect mathematical coupling.

#### Preoperative factors

Preoperative factors (independent variables) were age, sex, NQS in the operated limb, 6MWT, and LSI. Selection was made through theoretical reasoning based on previous research, research gaps, and factors considered likely to be important. To balance the risk of omitted-variable bias against the risk of model overfitting, we chose to include up to five preoperative factors. Recommendations regarding the required number of observations per variable vary considerably across the literature, ranging from 10 to 15 observations per variable to simulation studies suggesting that substantially fewer observations may be adequate for estimating regression coefficients in prespecified linear regression models [24, 25]. Nevertheless, the limited number of observations per variable in the present study increased the risk of overfitting and unstable coefficient estimates [24].

In general, quadriceps strength is dependent on age, sex, and body size [26–29]; therefore, these factors were considered likely to be important to be included. As mentioned in the introduction, previous research has shown inconsistent findings regarding associations between age, sex, BMI and quadriceps strength outcomes, and objective measures of physical function need to be included. We chose not to include BMI because we had to limit the number of variables in the regression models. Furthermore, quadriceps strength was already normalized by body weight, and body weight is present in the formula for BMI. However, BMI may still reflect biomechanical and metabolic factors not fully captured by normalizing strength by body weight. For objective measures of physical function, we chose preoperative NQS in the operated limb, LSI, and 6MWT. The main reason for including 6MWT but not the 30-second chair stand test (30CST) was the limited sample size. The 6MWT was also considered to provide information complementary to preoperative NQS, as it reflects broader aspects of physical function, including the combined effects of lower-limb muscle strength, pain, neuromuscular control, and cardiopulmonary fitness. In the analysis of changes in NQS, we omitted the preoperative NQS, as it is mathematically embedded in the outcome. Although preoperative pain may be an important preoperative factor and a potential source of residual confounding, it was not included because of the limited sample size.

### Measurements

#### Participant characteristics and patient-reported outcome measures

The participant characteristics collected to describe the study group were age, sex, height, weight, BMI, previous joint arthroplasty in hip and/or knee, and self-reported sufficient physical activity (≥ 150 min/week) [30]. Patient-reported outcomes were captured using the 42-item Knee Injury and Osteoarthritis Outcome Score (KOOS) [31, 32], and overall satisfaction was rated on a 4-point Likert scale 1 year after surgery.

#### Quadriceps strength, 6MWT, and 30CST

Quadriceps strength was measured as the maximum voluntary isometric contraction using a make test with a fixated MicroFET2 handheld dynamometer (Hoggan Scientific, Salt Lake City, UT, USA). The validity of this measurement is high [33] and its reliability is excellent [26]. Strength testing was performed using a dynamometer fixed against the lower-leg lever of a leg-extension apparatus (Leg Extension 4000, Ergo Fit, Pirmasens, Germany). The lower-leg lever and the dynamometer were positioned at the distal aspect of the lower leg, immediately proximal to the ankle. Individual machine settings, including seat and lever-arm positions, were recorded at baseline and replicated at the follow-up testing. Participants were seated with knees positioned at 90° flexion, arms folded across the chest, and the back supported against the backrest. They were instructed to exert their maximal voluntary isometric force for 5 s, while receiving verbal encouragement. Testing began with the operated limb and alternated between limbs with a 60-second rest period between tests. The procedure was repeated until up to three trials had been performed for each limb. A familiarization trial at low level effort was performed before testing at both baseline and follow-up. The highest value from each side was used for the analysis. Participants were asked about knee pain before strength testing and whether they perceived pain to limit their ability to generate maximal force during testing. The examiner also assessed whether pain appeared to substantially influence test performance. However, no standardized pain rating scale was recorded at the time of testing. 6MWT was performed according to guidelines from the Osteoarthritis Research Society International [19]. It was performed in a 20-m-long corridor, and the participant’s walking distance (m) in 6 min was recorded. Walking aids were permitted if required by the participant, and participants used their usual footwear during testing. 6MWT is used for various conditions, including TKA [20, 34]; it demonstrates good reliability [20, 35] and sensitivity to change [20, 35] following TKA. For the 30CST, a chair with a 44-cm seat height was used, and the number of sit-to-stand repetitions completed in 30 s was recorded. 30CST has excellent reliability [36] and is a valid measure of lower extremity physical function for people aged 60–89 years [37]. The order of testing was 30 CST, followed by 6MWT and quadriceps strength. Participants were allowed to rest between performance tests as needed. Rest periods were not standardized, apart from the 60-second rest interval between strength testing trials described above. No adverse events were reported during the preoperative or 1-year follow-up testing.

### Study size

The study included all individuals who met the inclusion criteria at baseline. Participants were consecutively enrolled over the 2-year study period. The final sample size reflected the eligible population available during the study period.

### Statistical methods

Continuous data were assessed for normality with the visual inspection of histograms and normality plots. Participant characteristics and baseline test results were summarized with descriptive statistics.

Three different models of standard multiple linear regression were used. All independent variables were selected based on theoretical reasoning and entered simultaneously. In the first model, NQS in the operated limb at 1 year after surgery was the dependent variable, and the independent variables were age, sex, preoperative NQS in the operated limb, 6MWT, and LSI. In the second model, changes in NQS in the operated limb from baseline to 1 year after surgery was the dependent variable, and the independent variables were age, sex, 6MWT, and LSI. In the third model, LSI at 1 year after surgery was the dependent variable, and the independent variables were age, sex, preoperative NQS in the operated limb, 6MWT, and LSI. To address concerns regarding model complexity relative to sample size, post hoc sensitivity analyses were performed for the models evaluating NQS at 1 year and LSI at 1 year. Reduced models including only preoperative NQS and preoperative LSI were fitted and compared with the corresponding prespecified models with respect to regression coefficients, adjusted *R*², and overall model significance.

Model assumptions were checked by inspecting the normal probability plot of the standardized residuals and the scatterplot of the standardized residuals [38] (Additional file 1). Standardized residuals exceeding ± 3 were considered indicative of potential outliers. In addition, Cook’s distance was examined to identify influential observations, with values > 1 considered indicative of substantial influence [38]. Scatterplots of each independent variable against the dependent variables were inspected to assess linearity, identify potential outliers, and evaluate the appropriateness of including them as linear terms in the regression models [39]. Multicollinearity between the independent variables was checked for using the Pearson correlation coefficient, tolerance and variance inflation factor. Correlations > 0.7, tolerance < 0.10, and variance inflation factor > 10 were considered indicative of potential multicollinearity [38, 39]. Participants were removed from the final analysis if they did not complete strength testing at 1 year after surgery. Regression coefficients were presented for both univariable and full model data. The univariable analyses were performed for descriptive purposes and were not used for predictor selection.

Attrition bias was tested for by conducting between-group analyses of baseline characteristics and baseline test results between the participants included in final analysis and those with incomplete data (*n* = 17) using the Mann–Whitney U test for continuous variables and the chi-square test or Fisher’s exact test for dichotomous variables. All statistical tests were performed using the Statistical Package for Social Sciences version 28.0 (SPSS, Inc., Chicago, IL, USA). Statistical significance was set at *p* < 0.05.

## Results

**Fig. 1:**
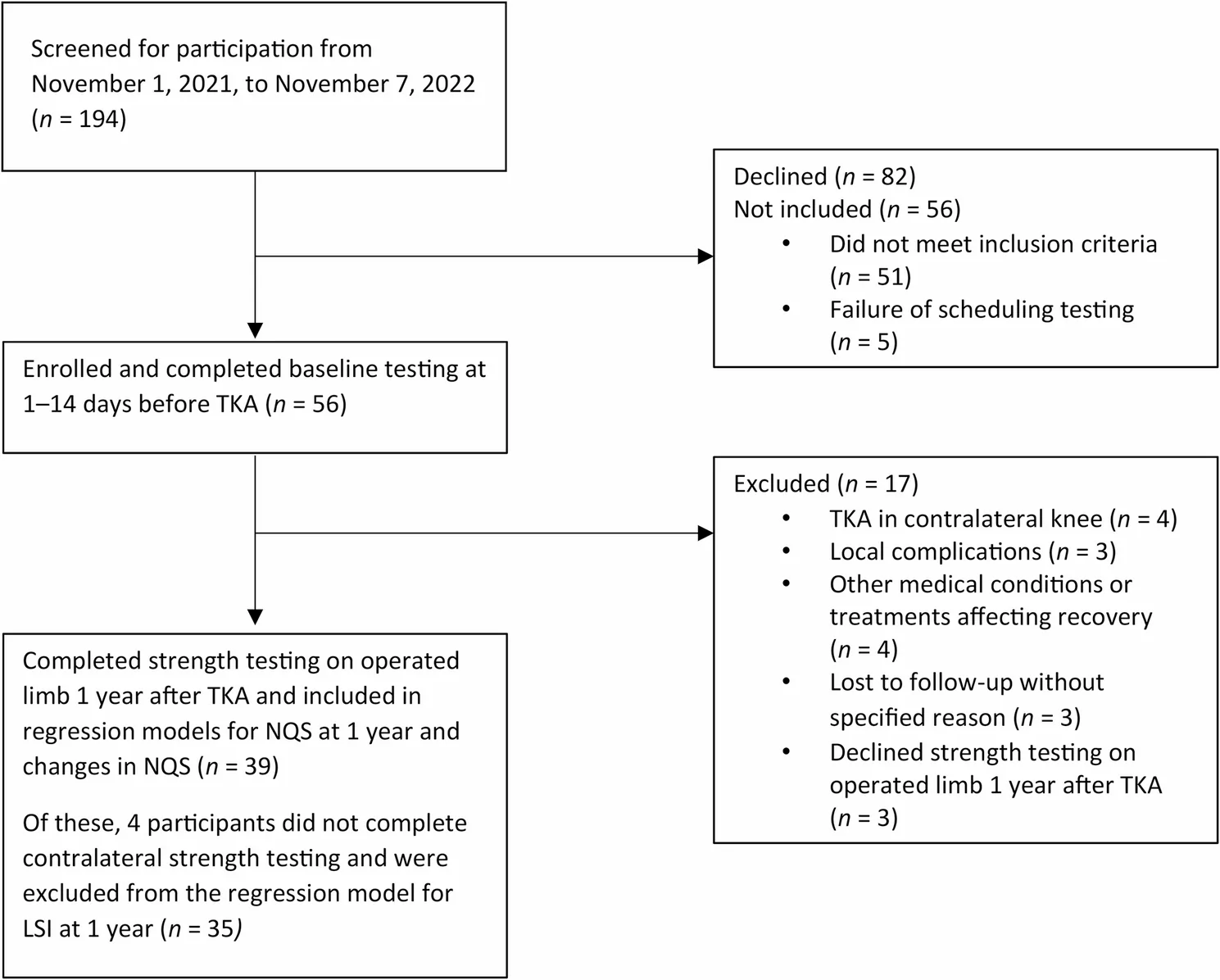
Study flow chart. TKA: total knee arthroplasty

One hundred and ninety-four participants were screened for participation. Of these, 82 declined, 51 did not meet the inclusion criteria, and five were excluded due to a failure to schedule preoperative testing. Finally, 56 participants consented to participate and completed baseline testing. Of these, 39 (70%) participants returned to the follow-up assessment 1 year after TKA, completed strength testing in the operated limb, and were included in final analysis. Four participants declined strength testing in the contralateral limb and were excluded from the analysis for LSI at 1 year after TKA, resulting in a sample of 35 participants for this analysis. A total of 17 participants were excluded from final analysis due to: TKA in the contralateral knee (*n* = 4), local complications following TKA (manipulation under anesthesia *n* = 2, complication with muscle attachment *n* = 1), other medical conditions or treatments affecting recovery (i.e., sepsis, arthrodesis of foot, breast cancer, and shoulder arthroplasty, *n* = 4), lost to follow-up without specified reason (*n* = 3), and declined strength testing on the operated limb at 1 year after TKA (*n* = 3). Figure 1 provides a flow chart for the study. In the attrition analysis, participants (*n* = 17) not included in the final analysis scored lower on preoperative KOOS–pain (*p* = 0.021), KOOS–activities of daily living (*p* = 0.015), and 30CST (*p* = 0.003) compared with those included in the final analysis (Table 1).

**Table 1:** Baseline characteristics, baseline test results and attrition analysis

Characteristic/test result	Included in final analysis, *n* = 39	Not included in final analysis, *n* = 17	Between-group comparison
Sex (women)	n = 15 (39%)	n = 7 (41%)	p = 1.000^a^
Age (years)	74 (4.7), 65–85	72 (5.4), 65–80	p = 0.106^b^
Height (cm)	173 (10), 145–188	173 (7.3), 163–188	p = 0.823^b^
Weight (kg)	84 (16), 53–125	85 (14), 67–122	p = 0.796^b^
BMI (kg/m^2^)	29 (3.3), 22–36	28 (3.4), 24–35	p = 0.769^b^
No previous arthroplasty in hip and/or knee	n = 28 (72%)	n = 11 (65%)	p = 0.830^a^
Sufficient physical activity(≥ 150 min/week)	n = 29 (74%)	n = 12 (71%)	p = 0.775^c^
NQS operated limb (%)	37 (8.6), 22–58	38 (11), 16–57	p = 0.702^b^
NQS contralateral limb (%)	44 (13), 23–83	41 (11), 23–66	p = 0.470^b^
LSI (%)	87 (16), 62–125	92 (17), 68–133	p = 0.306^b^
6MWT (m)	416 (113), 228–740	382 (97), 180–565*	p = 0.426^b^
30CST (n)	10 (3.5), 0–22	7.2 (3.7), 0–14	**p = 0.003** ^b^
KOOS—symptoms (0–100)	52 (16), 18–82	50 (17), 25–82	p = 0.707^b^
KOOS—pain (0–100)	50 (13), 19–89	41 (16), 22–86	**p = 0.021** ^b^
KOOS—ADL (0–100)	57 (14), 28–88	48 (15), 24–85	**p = 0.015** ^b^
KOOS—QoL (0–100)	27 (13), 0–56	21 (13), 0–44	p = 0.242^b^

Presenting baseline characteristics, baseline test results, and an attrition analysis comparing participants included in the final analysis and those who completed baseline testing but not strength testing 1 year after TKA. Values are presented as mean (SD), range (min–max) and n (%). Significant differences (*p* < 0.05) in bold. * *n* = 16 ^a^Chi-square test with continuity correction ^b^Mann–Whitney U test ^c^Fisher’s exact test due to 1 cell (25%) with an expected count less than 5. 6MWT: 6-minute walk test; 30CST: 30-second chair stand test; ADL: activities of daily living; BMI: body mass index; KOOS: Knee Injury and Osteoarthritis Outcome Score; LSI: limb symmetry index of quadriceps strength (operated limb divided by contralateral limb * 100); NQS: normalized quadriceps strength (strength divided by body weight * 100); QoL: quality of life; SD: standard deviation

The mean time to follow-up was 51.9 weeks after TKA (standard deviation (SD) 1.0, range 46–53). Table 1 presents the participant characteristics and baseline test results. One year after TKA, 32 (82%) participants rated overall satisfaction as “very satisfied” and seven (18%) participants gave a rating of “somewhat satisfied.” Seven (18%) participants had undergone TKA in the contralateral knee prior to study inclusion and were included in all regression analyses. The mean NQS in the operated limb was 37% (SD 8.6, 95% CI: 34 to 40) at preoperative testing and 46% (SD 11, 95% CI: 42 to 49) 1 year after TKA. The mean NQS in the contralateral limb was 44% (SD 13, 95% CI: 40 to 48) at preoperative testing. Among the 35 participants included in the regression analysis of LSI at 1 year after TKA, mean NQS in the contralateral limb was 45% (SD 13, 95% CI: 41 to 49) at preoperative testing and 49% (SD 13, 95% CI: 45 to 54) 1 year after TKA. Preoperative LSI values exceeded 100% in 10 of 39 participants (26%). Among the 35 participants included in the regression analysis of LSI at 1 year after TKA, 8 (23%) had preoperative LSI values above 100%, while 12 (34%) had LSI values above 100% at 1 year after TKA and the mean LSI at 1 year was 95% (SD 13, 95% CI: 91 to 100).

Univariable results are presented in Table 2, and results from the full multiple linear regression models are presented in Tables 3, 4 and 5. No evidence of problematic multicollinearity among the preoperative factors was detected (maximum VIF = 2.1, minimum tolerance = 0.48, maximum Pearson *r* = 0.58). Preoperative NQS showed little correlation with preoperative LSI (*r* = 0.085, *p* = 0.607), whereas stronger correlations were observed between preoperative NQS and contralateral limb NQS (*r* = 0.775, *p* < 0.001) and between preoperative LSI and contralateral limb NQS (*r* = − 0.537, *p* < 0.001). Visual inspection of the normal probability plots of the standardized residuals and the scatterplot of the standardized residuals revealed no major violations of model assumptions (Additional file 1: Figures S1–S3). Residuals appeared approximately normally distributed, no substantial heteroscedasticity was observed, and the relationships between preoperative factors and outcomes appeared reasonably linear. No extreme standardized residuals (absolute values > 3) or influential observations (maximum Cook’s distance: 0.25 for NQS 1 year, 0.28 for change in NQS, and 0.44 for LSI 1 year) were identified.

**Table 2:** Univariable unstandardized regression coefficients (*n* = 39)

Preoperative factor	NQS at 1 year (*n* = 39)		*p* value	Changes in NQS (*n* = 39)		*p* value		LSI at 1 year (*n* = 35)		*p* value
Age	-0.72 (-1.4 to -0.001)		0.050	-.22 (-0.70 to 0.26)		0.352		0.48 (-0.49 to 1.4)		0.320
Sex	**-10 (-16 to -3.4)**		**0.004**	-2.2 (-6.8 to 2.5)		0.350		0.54 (-8.8 to 9.9)		0.908
6MWT (per 100 m)	**6.2 (3.8 to 8.6)**		**<0.001**	1.8 (-0.18 to 3.7)		0.075		-1.7 (-5.7 to 2.4)		0.401
NQS operated limb	**0.96 (0.70 to 1.2)**		**<0.001**	-*		-*		-0.36 (-0.87 to 0.17)		0.175
LSI	-0.15 (-0.36 to 0.067)		0.173	**-0.19 (-0.32 to -0.066)**		**0.004**		**0.36 (0.11 to 0.61)**		**0.006**

Univariable unstandardized regression coefficients between the five preoperative factors and the three dependent variables presented with the corresponding 95% CI and p value. Significant coefficients and p values in bold. 6MWT: 6-minute walk test; LSI: limb symmetry index of quadriceps strength (operated limb divided by contralateral limb * 100); NQS: normalized quadriceps strength (strength divided by body weight * 100); TKA: total knee arthroplasty; * NQS was not included as preoperative factor in the model for changes in NQS

**Table 3:** Multiple linear regression analysis of normalized quadriceps strength in the operated limb 1 year after TKA (*n* = 39)

Preoperative factor	Unstandardized coefficient *B*	95% CI (*B*)	Standardized coefficient *β*	*p *value
Age	0.055	-0.44 to 0.55	0.024	0.821
Sex (0 = men, 1 = women)	-4.2	-8.7 to 0.32	-0.19	0.068
NQS operated limb	0.72	0.40 to 1.0	0.58	**<** **0.001**
6MWT (per 100 m)	2.3	-0.27 to 4.8	0.24	0.078
LSI	-0.17	-0.29 to -0.037	-0.25	**0.013**

Overall model *R*^2^ = 0.731, adjusted *R*^2^ = 0.690, *F* = 17.9, *p* < 0.001, *n* = 39. Significant p values (*p* < 0.05) for preoperative factors in bold. 6MWT: 6-minute walk test; LSI: limb symmetry index of quadriceps strength (operated limb divided by contralateral limb * 100); NQS: normalized quadriceps strength (strength divided by body weight * 100); TKA: total knee arthroplasty

**Table 4:** Multiple linear regression analysis of changes in normalized quadriceps strength in the operated limb from baseline to 1 year after TKA (*n* = 39)

Preoperative factor	Unstandardized coefficient *B*	95% CI (*B*)	Standardized coefficient *β*	*p* value
Age	0.047	-0.46 to 0.56	0.033	0.851
Sex (0 = men, 1 = women)	-2.6	-7.0 to 1.7	-0.19	0.220
6MWT (per 100 m)	1.0	-1.2 to 3.2	0.17	0.342
LSI	-0.19	-0.32 to -0.059	-0.45	**0.0** **06**

Overall model *R*^2^ = 0.278, adjusted *R*^2^ = 0.193, *F* = 3.28, *p* = 0.022, *n* = 39. Significant p values (*p* < 0.05) for preoperative factors in bold. 6MWT: 6-minute walk test; LSI: limb symmetry index of quadriceps strength (operated limb divided by contralateral limb * 100); TKA: total knee arthroplasty

**Table 5:** Multiple linear regression analysis of limb symmetry index of quadriceps strength at 1 year after TKA (*n* = 35)

Preoperative factor	Unstandardized coefficient *B*	95% CI (*B*)	Standardized coefficient *β*	*p* value
Age	0.28	-0.75 to 1.3	0.10	0.584
Sex (0 = men, 1 = women)	-1.2	-11 to 8.1	-0.048	0.783
NQS operated limb	-0.63	-1.3 to 0.021	-0.42	0.057
6MWT (per 100 m)	2.9	-2.3 to 8.1	0.25	0.262
LSI	0.41	0.15 to 0.68	0.52	**0.004**

Overall model *R*^2^ = 0.314, adjusted *R*^2^ = 0.196, *F* = 2.66, *p* = 0.043, *n* = 35. Significant p values (*p* < 0.05) for preoperative factors in bold. 6MWT: 6-minute walk test; LSI: limb symmetry index of quadriceps strength (operated limb divided by contralateral limb * 100); NQS: normalized quadriceps strength (strength divided by body weight * 100); TKA: total knee arthroplasty

In our model for NQS in the operated limb 1 year after TKA, preoperative NQS (unstandardized coefficient *B* = 0.72, 95% CI: 0.40 to 1.0, *p* < 0.001) and LSI (unstandardized coefficient *B* = − 0.17, 95% CI: − 0.29 to − 0.037, *p* = 0.013) were the only variables significantly associated with the outcome. The model achieved an overall adjusted *R*^2^ of 0.690, with *F* = 17.9, and *p* < 0.001. In the corresponding univariable analysis, sex, 6MWT, and preoperative NQS were significantly associated with the outcome. Findings were largely unchanged in a post hoc sensitivity analysis using a reduced model including only preoperative NQS and preoperative LSI. Preoperative NQS remained significantly associated with NQS at 1 year (unstandardized coefficient *B* = 0.72 vs. *B* = 0.99, both *p* < 0.001), as did preoperative LSI (unstandardized coefficient *B* = − 0.17, *p* = 0.013 vs. *B* = − 0.19, *p* = 0.004). Adjusted *R*² remained similar (0.690 vs. 0.675), and both models were statistically significant overall (*p* < 0.001).

In our model for changes in NQS in the operated limb from baseline to 1 year after TKA, preoperative LSI was the only variable significantly associated with the outcome (unstandardized coefficient *B* = − 0.19, 95% CI: − 0.32 to − 0.059, *p* = 0.006). The model achieved an overall adjusted *R*^2^ of 0.193, with *F* = 3.28, and *p* = 0.022. In our model for LSI at 1 year after TKA, preoperative LSI was the only variable significantly associated with the outcome (unstandardized coefficient *B* = 0.41, 95% CI: 0.15 to 0.68, *p* = 0.004). The model achieved an overall adjusted *R*^2^ of 0.196, with *F* = 2.66, and *p* = 0.043. Findings were largely unchanged in a post hoc sensitivity analysis using a reduced model including only preoperative NQS and preoperative LSI. Preoperative LSI remained significantly associated with LSI at 1 year (unstandardized coefficient *B* = 0.41, *p* = 0.004 vs. *B* = 0.43, *p* = 0.003), whereas the association between preoperative NQS and LSI at 1 year was similar in magnitude and remained non-significant (unstandardized coefficient *B* = − 0.63, *p* = 0.057 vs. *B* = − 0.43, *p* = 0.068). Adjusted *R*² was slightly higher in the reduced model (0.242 vs. 0.196), and both models remained statistically significant overall (*p* = 0.005 vs. *p* = 0.043). In the corresponding univariable analyses, LSI was the only variable significantly associated with changes in NQS and LSI at 1 year after TKA.

## Discussion

The aim of this exploratory study was to investigate preoperative factors associated with NQS, changes in NQS and LSI 1 year after TKA in patients with uncomplicated recovery. The main findings in this selected sample were that the preoperative NQS in the operated limb and LSI were the only preoperative factors significantly associated with NQS 1 year after TKA, while LSI was the only preoperative factor significantly associated with changes in NQS from baseline to 1 year after TKA and for LSI at 1 year after TKA. Demographic factors and other objective measures of physical function (i.e., 6MWT) were not independently associated with any of the outcomes. These findings suggest that, in patients with uncomplicated recovery, preoperative NQS and LSI may be more strongly associated with NQS outcomes 1 year after TKA than general functional capacity or demographic characteristics. The findings should be considered exploratory and confirmation in larger adequately powered studies is warranted.

In the univariable analysis for NQS 1 year after TKA, sex and preoperative 6MWT were significantly associated with the outcome. However, these associations did not remain in the full model, indicating that their univariable effects were largely explained by stronger covariates. Conversely, LSI was not significant in the univariable analysis but became significant in the full model. As the effect estimate changed only marginally after adjustment, this finding may reflect increased precision of the estimate after accounting for the other covariates. The coefficient in the full model should therefore be interpreted as a conditional association. The full model explained 69% of the variance in NQS 1 year after TKA within the present sample (adjusted *R*^2^ = 0.690). The relatively high adjusted *R*^*2*^ may partly reflect sample-specific associations and should not be interpreted as evidence of strong predictive performance in other populations. Within this model, better preoperative NQS in the operated limb and a lower LSI were significantly associated with higher NQS 1 year after TKA. Based on the standardized coefficients β, preoperative NQS showed a stronger association with NQS 1 year after TKA (β = 0.58) compared with preoperative LSI (β = −0.25). When interpreting the unstandardized regression coefficients, a 5%-point higher preoperative NQS was associated with an estimated 3.6%-point higher NQS 1 year after TKA (95% CI: 2.0 to 5.0). Likewise, a 10%-point lower preoperative LSI was associated with an estimated 1.7%-point higher NQS 1 year after TKA (95% CI: 0.4 to 2.9).

The results from the full model for NQS 1 year after TKA indicate that preoperative NQS had the strongest association with NQS 1 year after TKA. Current recommendations for the management of KOA and for improving functional outcomes after TKA include exercise, physical activity, and weight reduction [13]. However, the observed association between preoperative NQS and NQS at 1 year does not demonstrate that increasing preoperative NQS will improve the postoperative outcome. The effectiveness of such interventions was not evaluated in the present study and should be evaluated in adequately powered interventional studies. Weight reduction has been associated with improved functional outcomes [40, 41], and reduced risk of postoperative infection and arthroplasty revision after TKA [42], and it may also contribute to higher NQS. The effect of prehabilitation is under debate, but is still generally recommended [43, 44].

Preoperative LSI was the only preoperative factor significantly associated with the outcome in both the univariable and full models for changes in NQS in the operated limb and LSI at 1 year after TKA. A lower preoperative LSI (in most cases a relatively lower NQS in the operated limb compared to the contralateral limb) was associated with greater changes in NQS, which aligns with previous research indicating that interlimb differences are reduced at 1 year after TKA [6]. In contrast, a higher preoperative LSI was associated with a higher LSI at 1 year after TKA. The interpretation of the association between LSI and changes in NQS should be made with caution. One hypothesis is that lower preoperative LSI may reflect a greater physiological potential for recovery due to mechanisms such as altered limb loading, reduced physical activity, and pain-related or arthrogenic muscle inhibition. However, this interpretation remains speculative, as the observed association may also be influenced by greater mathematical room for improvement, regression to the mean and mathematical coupling between NQS and LSI. All findings involving LSI should be interpreted in the light of the fact that LSI reflects the relationship between NQS in the operated and contralateral limbs rather than NQS in the operated limb alone. LSI values may exceed 100%, indicating higher NQS in the operated limb than in the contralateral limb. Improved LSI may reflect recovery of the operated limb, changes in the contralateral limb, or a combination of both. Furthermore, LSI values close to or above 100% do not necessarily indicate adequate bilateral NQS, as similar NQS values may reflect either high or low NQS in both limbs.

A low proportion of the variance was explained by the full models for changes in NQS (adjusted *R*² = 0.193) and LSI at 1 year after TKA (adjusted *R*² = 0.196). These findings suggest considerable variability in the outcomes that was not explained by the variables included in these two regression models. For instance, postoperative pain and the amount of physical activity, rehabilitation content, and exercise adherence together with psychosocial factors such as motivation, self-efficacy, social support and fear of movement may have contributed to postoperative NQS alterations.

Female sex has been reported as a negative predictor for quadriceps strength 1 year after TKA [8] and 1 year after surgically treated lower extremity fractures [45]. However, neither of these studies accounted for presurgical quadriceps strength. In our study, sex was not significantly associated with NQS 1 year after TKA (*B* = -4.2, 95% CI: -8.7 to 0.32, β = − 0.19, *p* = 0.068). Given the wide confidence interval, the estimate should be interpreted with caution and is compatible with both little or no association and a potentially meaningful negative association. Future studies with an adequate sample size are required to further explore this possible association.

### Limitations

There are several limitations to consider when interpreting the findings of this study. Patients were recruited over the study period and constituted the final study population. No a priori sample size calculation based on regression modeling was performed, which limits our ability to exclude the possibility of a type II error, meaning that important associations may have remained undetected. The relatively small sample size also limited the number of preoperative factors that could be included in our analyses, and interaction analyses were not feasible. Already, with five preoperative factors (independent variables) for 39 observations, there was a risk of model overfit [24]. However, preoperative factor selection was based on theoretical reasoning and previous research, rather than through data-driven selection [24, 25]. Including key demographic and functional variables was considered important to reduce the risk of omitted-variable bias, which can distort regression coefficients more than modest overfitting in small samples. Although no major violations of model assumptions were visually identified (Additional file 1: Figures S1–S3) and post hoc sensitivity analyses yielded largely consistent results, the limited number of observations per independent variable may have reduced the precision of the observed associations. The magnitude of the observed associations and their statistical significance may still be sensitive to small changes in the sample or alternative model specifications that were not evaluated. Therefore, the observed associations are better viewed as exploratory rather than precise estimates. Furthermore, multiple exploratory analyses were performed across several outcomes and preoperative factors, increasing the probability of chance findings. An additional limitation is the mathematical dependency between NQS, changes in NQS and LSI, as these measures are calculated from shared NQS measurements.

It is likely that the study attracted participants with better overall health and functional performance than the 82 individuals that declined. The postoperative exclusion criteria were prespecified and were chosen to ensure that follow-up strength measurements reflected recovery after TKA as accurately as possible. However, these exclusions may have introduced selection bias. *Post hoc* analyses indicated attrition bias, as participants with complete baseline data that were not included in the final analysis (*n* = 17) had more pain, worse self-reported function, and lower 30CST performance than those included. Although there were no between-group differences for the analyzed preoperative factors, the final study sample may represent a healthier subgroup with a more favorable recovery trajectory than the broader TKA population.

Preoperative pain, BMI, patient-reported outcome measures (e.g., KOOS), radiographic appearances, type of prosthesis, kinesiophobia, and comorbidities are examples of potentially relevant preoperative factors that were not included in our analyses and may represent sources of residual confounding. Even though different rehabilitation strategies have shown comparable outcomes after TKA [17, 43, 46–48], the absence of detailed information on exercise adherence, physiotherapy intensity, load progression, physical activity, and rehabilitation content throughout the first postoperative year is another limitation to this study. Additionally, pain in the postoperative period may influence postoperative strength gains. These factors were not included because the aim of the study was to investigate preoperative factors.

Another limitation is that quadriceps strength was assessed using isometric contractions at 90° knee flexion. Although this method is clinically feasible and has good reliability and validity, it may not fully reflect the dynamic functional capacity of the quadriceps. Furthermore, pain during strength testing was not assessed using a standardized pain measure. Finally, the assessor was not blinded to baseline test results, and the study was retrospectively registered four months after participant enrollment had commenced.

### Generalizability

Considering the limitations of this study, some caution must be taken when generalizing its findings before they are confirmed by additional studies. The analyses were conducted in a selected sample with an uncomplicated recovery and complete one-year follow-up. The generalizability is greatest for patients 65 years and older who are treated with TKA in a fast-track care model, have uncomplicated recovery, and utilize standard primary care rehabilitation. The findings may be less applicable to patients with postoperative complications, persistent pain, poorer baseline function, patients undergoing revision TKA, patients with rheumatoid arthritis or neurological disorders, and individuals residing in nursing homes. Furthermore, caution is warranted when generalizing the findings to healthcare systems outside Sweden, as differences in healthcare organization, access to rehabilitation services, and postoperative care pathways may influence recovery after TKA.

## Conclusions

In this selected and relatively small sample, greater NQS in the operated limb and higher LSI 1 year after TKA appeared to be primarily associated with higher preoperative NQS in the operated limb and higher preoperative LSI, respectively. Greater NQS gains appeared to be associated with lower preoperative LSI, although this finding should be interpreted with extra caution. Age, sex, and 6MWT were not significantly associated with postoperative NQS, NQS gains, or LSI 1 year after TKA. The findings should be considered exploratory and primarily applicable to older adults with uncomplicated recovery after TKA. Confirmation in larger adequately powered studies is warranted.

## Supplementary Information

Below is the link to the electronic supplementary material.

Supplementary Material 1

## Data Availability

The data that support this study’s findings are available from the corresponding author upon reasonable request. The data are not publicly available due to ethical and legal restrictions.

## Abbreviations

30CST: 30-second chair stand test
6MWT: 6-minute walk test
ADL: Activities of daily living
BMI: Body mass index
KOA: Knee osteoarthritis
KOOS: Knee Injury and Osteoarthritis Outcome Score
LSI: Limb symmetry index
N: newton
NQS: Normalized quadriceps strength
QoL: Quality of Life
SD: Standard deviation
TKA: Total knee arthroplasty
