# Supplementary material for: Preoperative factors associated with quadriceps strength 1 year after total knee arthroplasty: a prospective longitudinal study
Source: BMC Musculoskelet Disord. 2026 Oct 21;27:914. doi: 10.1186/s12891-026-10657-8 (PMC13700487; doi:10.1186/s12891-026-10657-8)
Supplement: Supplementary file 1 [file 12891_2026_10657_MOESM1_ESM.docx]

Additional file 1

**Figure S1**. Normal probability plot and scatterplot for the standardized residuals from the multiple linear regression for normalised quadriceps strength at 52 weeks after surgery.

**Figure S2**. Normal probability plot and scatterplot for the standardized residuals from the multiple linear regression for changes in normalized quadriceps strength from baseline to 52 weeks after surgery.

**Figure S3**. Normal probability plot and scatterplot for the standardized residuals from the multiple linear regression for Limb Symmetry Index (LSI) at 52 weeks after surgery.
